# Epidemiological and clinical characteristics associated with enterovirus D68 respiratory diseases in Asian children: a systematic review and meta-analysis

**DOI:** 10.1016/j.jped.2025.101475

**Published:** 2025-11-14

**Authors:** Xiaojing Lu, Bingbing Li, Shufang Li, Lihong Wang, Guangen Guo, Yanli Zhang, Jiajia Duan, Changlian Zhu

**Affiliations:** a The Third Affiliated Hospital of Zhengzhou University, Department of Pediatrics, Division of Pulmonology, Zhengzhou, Henan, China; b The Third Affiliated Hospital and Institute of Neuroscience of Zhengzhou University, Henan Key Laboratory of Child Brain Injury and Henan Pediatric Clinical Research Center, Zhengzhou, Henan, China; c The Third Affiliated Hospital of Zhengzhou University, Department of Neonatology, Zhengzhou, Henan, China; d University of Gothenburg, Institute of Neuroscience and Physiology, Sahlgrenska Academy, Center for Brain Repair and Rehabilitation, Gothenburg, Sweden; e Karolinska Institutet, Department of Women’s and Children’s Health, Stockholm, Sweden

**Keywords:** EV-D68, Epidemiology, Clinical characteristics, Children, Meta-analysis

## Abstract

**Objective:**

This study aimed to determine the epidemiology and clinical characteristics of EV-D68-associated respiratory diseases among Asian children.

**Sources:**

The PubMed, Embase, Web of Science, Scopus, China National Knowledge Infrastructure (CNKI), VIP, WanFang, and SinoMed electronic databases were searched from inception to February 28, 2025, to identify relevant articles. Studies reporting the detection rate of enterovirus D68 (EV-D68) in pediatric patients with respiratory tract infections were included. The methodological quality regarding the risk of bias was assessed according to the approach proposed by the Joanna Briggs Institute.

**Summary of the findings:**

Twenty studies involving 47,451 participants were included; additionally, 450 participants were positive for EV-D68 infection. The detection rate of EV-D68 ranged from 0.23 % to 10.56 %, with a pooled prevalence of 1.29 % (95 % CI 0.88–1.78 %), but significant heterogeneity (*I*² = 94.20 %, *p* < 0.001) indicated that this estimate was context-dependent. Subgroup analyses revealed that detection rates varied substantially by country, study period, and income level. Pneumonia is the most common respiratory disease presentation. EV-D68 is most prevalent during the summer and autumn.

**Conclusion:**

This is the first review to focus specifically on Asian children, which suggests that EV-D68 detection rates vary significantly across Asian populations and are especially influenced by geographic location and surveillance timing. EV-D68 infection in children is most frequently associated with pneumonia. These findings support targeted regional surveillance networks during the summer-autumn seasons to improve clinical diagnostics and public health responses.

**Trial Registration:**

PROSPERO Identifier: CRD42023449889.

## Introduction

Enterovirus D68 (EV-D68), a member of the Picornaviridae family, has reemerged as a significant pediatric respiratory pathogen. Unlike most enteroviruses, EV-D68 is isolated from respiratory samples (specifically from the nose), and its biological and molecular characteristics are more similar to those of rhinoviruses. EV-D68 was initially identified in 1962 and gained global attention during the 2014 North American outbreak, which linked it to severe respiratory illness and acute flaccid myelitis (AFM) [1]. In recent years, the volume of epidemiological data related to EV-D68 has gradually increased [2, 3, 4, 5]. The detection rate of EV-D68 varies widely throughout the world [6]. While western epidemiology is well characterized, Asian data remain fragmented, obscuring region-specific rates, clinical profiles, and seasonal trends. EV-D68 is most prevalent in children. Children aged < 16 years account for > 90 % of all EV-D68 infections worldwide [7]. Thus, the authors performed a systematic review and meta-analysis of the published literature to summarize the current knowledge of EV-D68-associated respiratory diseases in Asian children with respect to the detection rate, clinical characteristics, seasonality, and rate of coinfections.

## Methods

### Search strategy

The authors conducted a systematic review and meta-analyses following the Preferred Reporting Items for Systematic Review and Meta-Analyses (PRISMA) 2020 guidelines [8]. The review protocol was registered in PROSPERO (CRD42023449889). This meta-analysis was a secondary study based on previously published data; therefore, this study received an exemption from the Ethics Committee of the Third Affiliated Hospital of Zhengzhou University.

The authors searched the PubMed, Embase, Web of Science, Scopus, China National Knowledge Infrastructure (CNKI), VIP, WanFang, and SinoMed electronic databases for studies published up to February 28, 2025, with no language restrictions (Supplementary Table 1).

### Inclusion and exclusion criteria

Studies were included if they met the following criteria: (1) they reported the detection rate of EV-D68 in pediatric patients (aged < 18 years) with respiratory tract infections across Asian populations; (2) they confirmed EV-D68 infection via reverse transcription–polymerase chain reaction (RT-PCR) in respiratory specimens; (3) they had sufficient detailed and extractable data to characterize the epidemiological patterns and clinical manifestations associated with EV-D68 infections; and (4) they included studies conducted over at least a full calendar year. Reviews, case reports, conference papers, notes, editorials, and nonobservational research were excluded. Studies in which EV-D68 was detected by the following methods (including viral culture, antigen detection, or antibodies) were excluded.

### Literature screening and data extraction

The authors used EndNote to manage the search output. Two reviewers (L.X. and W.L.) independently screened the titles and abstracts of the studies and assessed their eligibility. After screening the published articles for eligibility, relevant data and information, including the first author’s name, year of publication, study design, sampling method, time of sample collection, country, study period, age group, EV-D68 diagnostic method, sample type, sample size, number of EV-D68-positive cases, basic information, demographic information, and clinical symptoms, were extracted from each eligible study and curated in a WPS Excel sheet. For studies that included both adults and children, only data related to children were extracted. Two authors (L.X. and W.L.) independently extracted and evaluated the data from the eligible studies. Any inconsistencies or disagreements were discussed with a third author (L.B.) and resolved via consensus.

### Quality assessment

The Joanna Briggs Institute quality assessment tool was employed to evaluate the quality of the studies that were included in the final analysis [9]. The scoring of individual studies was conducted according to frequency scales, with scores based on responses of yes, no, unclear, or not applicable. To calculate the total quality score for each study, the authors utilized the total number of positive scores.

### Statistical analysis

For binary outcomes, proportional meta-analyses with 95 % confidence intervals were conducted via Stata 12.0 (StataCorp LP). The "metaprop" package was employed to implement double arcsine transformations on raw proportions prior to pooling, ensuring that normality assumptions were satisfied. Between-study heterogeneity was quantified through Cochran's Q test (χ²) and Higgins's *I²* metrics, with *I²* thresholds interpreted as follows: ≤ 25 % (negligible), 26–50 % (moderate), and > 50 % (substantial). Given the anticipated variations in clinical populations and research methodologies across studies, all analyses adopted DerSimonian–Laird random effects models to account for both within-study and between-study variance components. The authors further performed subgroup-level meta-analysis to address heterogeneity. The subgroups were based on the timing of sample collection, country, country income level, sex, age group, period of study (before and after 2014), and EV-D68 diagnostic method. Begg’s test and funnel plots were used to assess publication bias, with a p value < 0.05 and skewness of the funnel diagram indicating the presence of publication bias.

## Results

### Study search results

**Fig. 1:**
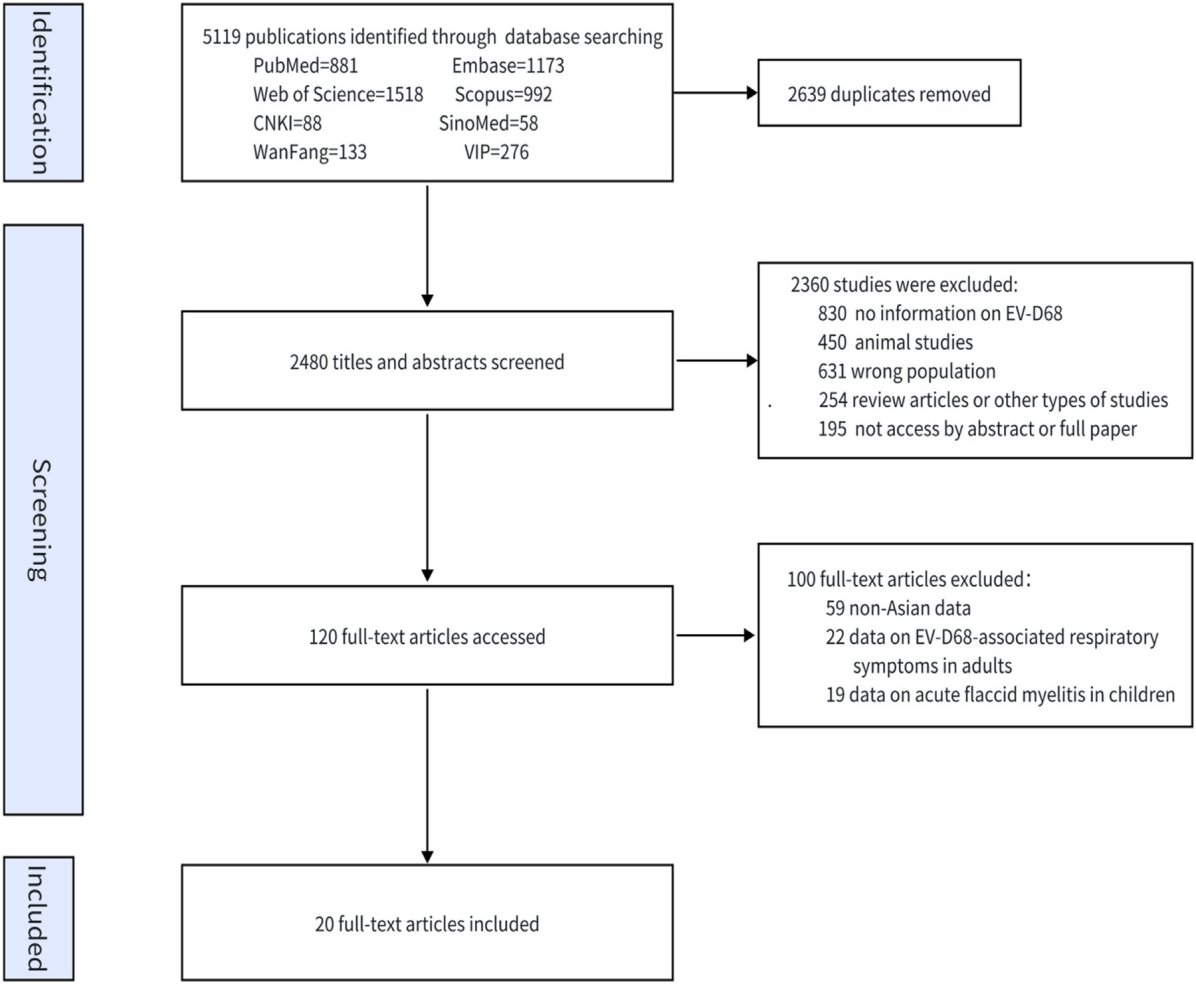
Flowchart of study selection.

In total, 5119 studies were screened for eligibility. Of these, 120 full texts were reviewed, and 20 studies were ultimately included for analysis (Fig. 1) [10, 11, 12, 13, 14, 15, 16, 17, 18, 19, 20, 21, 22, 23, 24, 25, 26, 27, 28, 29]. All the studies were written in English. Among the 20 included studies (Supplementary Table 2), 12 were retrospective. Eight studies were conducted in China, 5 were conducted in Japan, 3 were conducted in the Philippines, 2 were conducted in Thailand, 1 was conducted in Iran, and 1 was conducted in Myanmar. Five studies were performed in high-income countries, 11 were performed in upper-middle-income countries, and 4 were performed in lower-middle-income countries. The predominant technique used in these studies was RT-PCR. Four studies exclusively enrolled children younger than five years old.

A total of 47,451 participants were included in these 20 articles. Among 8768 children from 7 studies reporting age data, the overall median age was 0.92 years (IQR: 0.75–1.40). Gender data were reported in seven articles, with a male-to-female ratio of 1.50:1 (95 % CI 55.7–64.1 %) (Supplementary Table 2).

### Quality of the included studies

Based on the Joanna Briggs Institute quality evaluation checklist, the articles that were included in the final analysis had a mean quality score of 7.85, with scores ranging from six to nine. One study was a high-quality study, and the remaining studies were moderate-quality studies (Supplementary Table 3).

### Detection rates and subgroup analysis

**Fig. 2:**
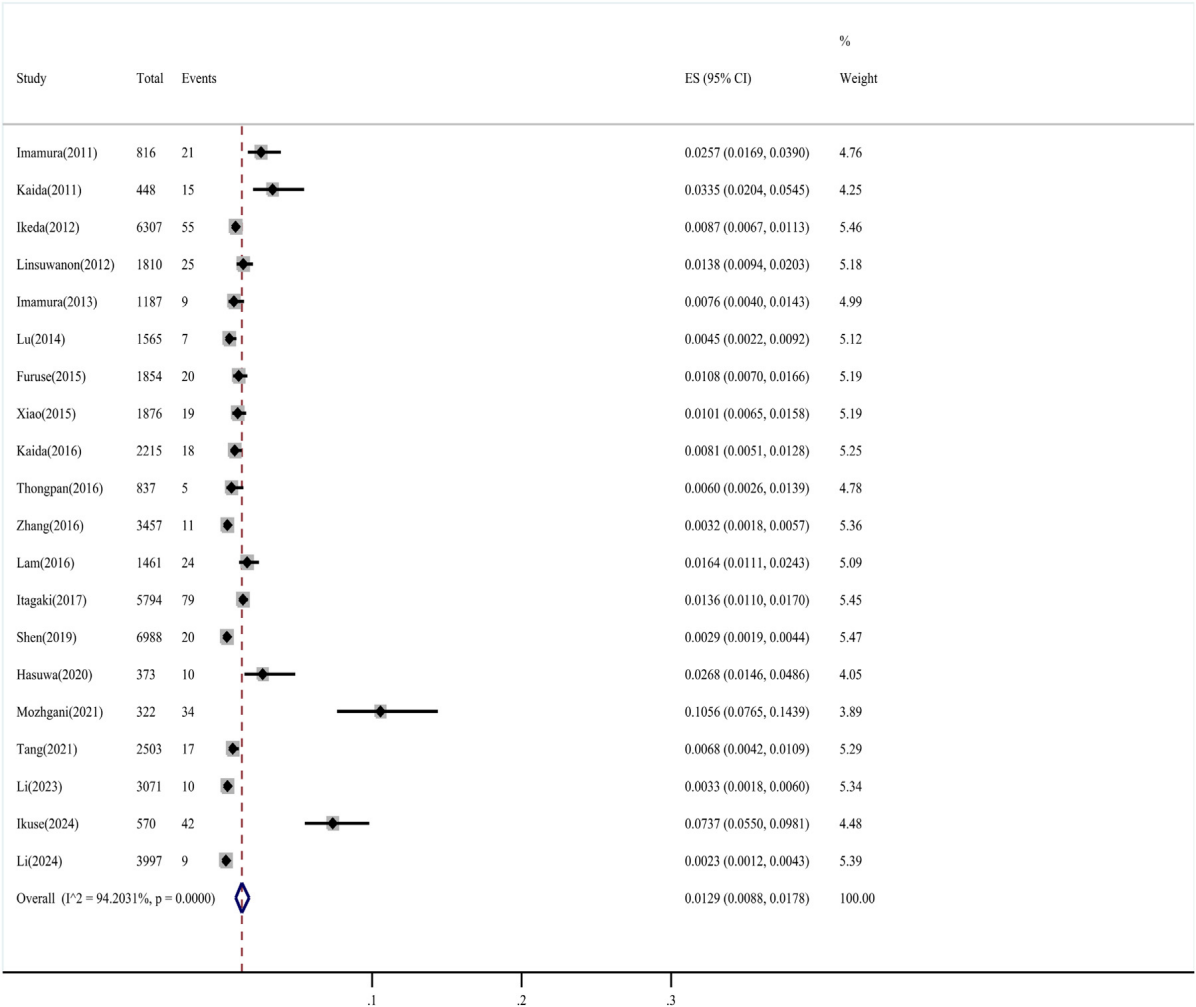
Forest diagram of the pooled prevalence estimates.

A total of 450 EV-D68 infections were reported among 47,451 participants. The detection rate of EV-D68 ranged from 0.23 % to 10.56 % in the included studies, with a pooled prevalence (random-effects model) of 1.29 % (95 % CI 0.88–1.78 %), but significant heterogeneity (I² = 94.20 %, *P* < 0.001) indicated that this estimate was context-dependent (Fig. 2).

Subgroup analyses (Table 1) revealed that detection rates varied substantially by country (highest in Iran [10.56 %] and Myanmar [7.37 %]), study period (3.02 % post-2014 vs. 1.18 % pre-2014), and income level (2.39 % in high-income countries). The high detection in Iran and Myanmar derives from single-study estimates requiring cautious interpretation.

**Table 1:** Subgroup analyses of factors associated with variability in EV-D68 detection among Asian children.

Subgroup classification	Detection rate ( %) (95 % CI)	N Studies	N Participants	Event	*I2* ( %)	*p* heterogeneity	*p* difference subtypes
Timing of samples collection							0.219
Prospectively	1.69 (0.90–2.72)	8	15,799	170	93.80	<0.001	
Retrospectively	1.09 (0.621–1.68)	12	31,652	280	94.76	<0.001	
Country							<0.001
Philippines	1.34 (0.58–2.38)	3	3857	50	NE	NE	
Japan	1.41 (0.89–2.05)	5	15,137	177	84.61	<0.001	
Thailand	1.10 (0.731–1.54)	2	2647	30	NE	NE	
China	0.53 (0.31–0.80)	8	24,918	117	85.13	<0.001	
Iran	10.56 (7.65–14.39)	1	322	34	NE	NE	
Myanmar	7.37 (5.50–9.81)	1	570	42	NE	NE	
Country income level							0.158
Lower-middle-income economies	1.41 (0.89–2.05)	4	4427	92	84.61	<0.001	
High-income economies	2.39 (0.69–5.05)	5	15,137	177	95.34	<0.001	
Upper-middle-income economies	0.90 (0.49–1.41)	11	27,887	181	93.51	<0.001	
Gender							0.912
Male	2.40 (1.07–4.20)	7	5088	81	91.34	<0.001	
Female	2.51 (0.854–4.93)	7	3012	64	91.43	<0.001	
Age range							0.108
Birth–18 years	1.07 (0.64–1.60)	14	34,239	301	95.57	<0.001	
Birth–5 years	3.50 (0.68–8.25)	4	1980	64	97.59	<0.001	
Study period							0.169
Before-2014	1.18 (0.83–1.58)	10	18,161	200	78.62	<0.001	
Post-2014	3.02 (0.64–6.99)	4	10,951	106	98.45	<0.001	
Diagnostic method							0.231
Classical RT-PCR	1.53 (0.97–2.21)	12	28,860	320	93.73	<0.001	
Real-Time RT-PCR	0.99 (0.45–1.72)	8	18,591	130	94.41	<0.001	

In the subgroup analysis, there were no heterogeneous results in three or fewer articles. NE = not estimable.

The detection rate of EV-D68 was greater in prospective studies and studies that included participants aged < 5 years, although the difference was not statistically significant. In the subgroup analysis based on sex, there was no significant difference in the detection rate between the male group and the female group (*p* = 0.912).

### Year-to-year circulation patterns

The authors included 20 articles, and 17 articles that provided detailed annual detection rates were analyzed to derive annual detection rates to understand the EV-D68 cycle pattern from year to year. The annual detection rates of EV-D68 among Asian children with respiratory infections from 2007 to 2019 fluctuated significantly, with intermittent peaks observed in 2008 (2.57 %), 2010 (2.15 %), 2014–2015 (1.14 %–1.68 %), and notably 2018 (4.07 %). The highest rate occurred in 2018, suggesting that a major outbreak occurred in that year (Supplementary Fig. 1).

### Demographic and clinical characteristics of EV-D68 patients

The total number of EV-D68 infections in the eligible studies was 450. The ages ranged from 0 to 18 years. Among the 372 EV-D68-positive children from 17 studies reporting age data, the overall median age was 3.50 years (IQR: 2.67–5.20). A total of 79.53 % (95 % CI 64.10–91.9 %) of the children were < 5 years, 29.24 % (95 % CI 18.72–40.85 %) were 5–14 years old, and 7.94 % (95 % CI 0.54–20.25 %) were older than 14 years. The male-to-female ratio of EV-D68-infected patients was 1.41:1 (95 % CI 51.85–64.88 %). Nine studies reported the comorbidity of pediatric EV-D68 infection patients. A total of 60 of 194 patients (22.25 %, 95 % CI 7.85–40.4 %) had at least one comorbidity. Among them, the most common comorbidities were asthma or recurrent wheezing (20.53 %, 95 % CI 4.33–42.72 %) (Table 2, Supplementary Table 4).

**Table 2:** Demographics and comorbidities of children with EV-D68 analyzed by meta-analysis.

**Variable**	**N Studies**	**n**	**prevalence**	**95% CI**	**Heterogeneity tests**			
					***Q***	***I^2^***	***t^2^***	***p***
**Demographics (%)**								
<5 years	14 b	194	79.53	64.10–91.9	87.67	85.17	0.3	<0.001
6–14 years	10	62	29.24	18.72–40.85	23.49	61.68	0.08	0.0052
15–18 years a	2	3	7.94	0.54–20.25	NE	NE	NE	NE
Male	17	231	58.43	51.85–64.88	24	33.33	0.02	0.09
**Comorbidities (%)**								
Asthma or recurrent Wheezing	8	51	20.53	4.33–42.72	52.64	86.7	0.34	<0.001
Cardiovascular disease a	2	3	6.91	0.1–19.4	NE	NE	NE	NE
Neurological disease a	1	3	17.64	NE	NE	NE	NE	NE
Gastrointestinal disease a	1	1	11.11	NE	NE	NE	NE	NE

*Q,* Cochran’s Q statistic for heterogeneity*; I*^2^*, I*^2^ index for the degree of heterogeneity; *t*^2^*,* tau-squared measure of heterogeneity; NE, not estimable.

a Data from two or fewer studies.

b 14 comprises four studies that exclusively involved this age group and ten additional studies that included EV-D68-positive children under 5 years of age within broader cohorts.

A total of 174 of 251 patients (77.62 %, 95 % CI 58.43–92.68 %) across 15 studies were diagnosed with pneumonia, which is the most common respiratory disease presentation. This was followed by upper respiratory tract infection (URTI), which was diagnosed in 126 of 235 patients (45.22 %, 95 % CI 27.16–63.90 %) across 7 studies. Other respiratory diseases included asthma, bronchitis (33.12 %), bronchial asthma (19.67 %), bronchiolitis (10.83 %), and bronchitis (8.46 %). The most prevalent clinical symptoms were cough (92.09 %, 95 % CI 78.35–99.82 %), fever (61.09 %, 95 % CI 45.48–75.75 %), wheezing (53.09 %, 95 % CI 40.80–65.21 %), sputum production (47.61 %, 95 % CI 32.19–63.24 %), dyspnea (42.14 %, 95 % CI 20.86–64.84 %), and runny nose (30.8 %, 95 % CI 14.04–50.22 %). The less frequent symptoms were chestache (12.85 %), sore throat (12.27 %), vomiting (10.19 %), sneezing (6.93 %), and diarrhea (6.67 %) (Table 3, Supplementary Table 5).

**Table 3:** The clinical characteristics and seasonality of children with EV-D68 analyzed by meta-analysis.

**Variable**	**N Studies**	**n**	**prevalence**	**95% CI**	**Heterogeneity tests**			
					***Q***	***I^2^***	***t^2^***	***p***
**Diagnosis (%)**								
URTI a	7	126	45.22	27.16–63.9	44.09	86.39	0.2	<0.001
Pneumonia a	15	174	77.62	58.43–92.68	131.41	89.35	0.5	<0.001
Asthmatic bronchitis	4	31	33.12	5.15–69.34	39.69	92.44	0.48	<0.001
Bronchial asthma	7	41	19.67	8.18–34.02	27.21	77.95	0.12	<0.001
Bronchiolitis	3	13	10.83	5.07–18.02	1.37	0	0	0.5039
Bronchitis	5	17	8.46	4.5–13.29	1.31	0	0	0.86
**Symptoms (%)**								
Fever	13	170	61.09	45.48–75.75	68.12	82.38	0.22	<0.001
Cough	13	228	92.09	78.35–99.82	89.45	86.58	0.33	<0.001
Sputum production	5	35	47.61	32.19–63.24	6.58	39.19	0.04	0.16
Dyspnea	10	79	42.14	20.86–64.84	68.89	86.93	0.39	<0.001
Wheezing	16	167	53.09	40.8–65.21	70.7	78.79	16.2	<0.001
Chest ache	3	5	12.85	2.8–26.97	0.406	0	0	0.816
Sore throat	4	9	12.27	1.45–28.9	6.23	52.04	0.08	0.1
Runny nose	8	84	30.8	14.04–50.22	40.89	82.88	21.65	<0.001
Sneezing b	2	2	6.93	0–20.86	NE	NE	NE	NE
Vomiting b	2	3	10.19	0.05–28.82	NE	NE	NE	NE
Diarrhea	7	12	6.67	0.83–15.81	14.98	59.93	0.07	0.02
**Clinical outcomes (%)**								
ICU admission	12	31	11.55	1.49–26.86	70.14	84.32	0.32	<0.001
Fatality rate	15	7	0.91	0–3.32	8.85	0	0	0.84
**Seasonality (%)**								
Spring	8	26	16.84	9.82–5.05	8.88	21.15	0.015	0.26
Summer	13	109	43.83	26.60–61.79	1113.35	89.41	0.34	<0.001
Autumn	18	235	45.15	26.82–64.12	236.64	92.82	0.54	<0.001
Winter	9	70	34.85	11.63–0.62	102.13	92.17	0.56	<0.001

*Q,* Cochran’s Q statistic for heterogeneity; *I*^2^*, I^2^* index for the degree of heterogeneity; *t*^2^*,* tau-square measure of heterogeneity; NE, not estimable; URTI, upper respiratory tract infection.

a proportions for pneumonia/URTI are derived from different denominators across studies and are not additive.

b Data from two or fewer studies.

A total of 31 of the 203 patients across the seven studies were transferred to the ICU (11.55 %, 95 % CI 1.49–26.86 %), and among the 248 patients, 7 died, for an overall fatality rate of 0.91 % (95 % CI 0–3.32 %) (Table 3, Supplementary Table 5).

### Seasonality of EV-D68 infections

Among the 18 studies, 45.15 % (95 % CI 26.82–64.12 %) of the EV-D68 infections were detected in autumn, followed by summer (43.83 %, 95 % CI 26.60–61.79 %) across 13 studies, winter (34.85 %, 95 % CI 11.63–62.00 %) in 9 studies, and spring (16.84 %, 95 % CI 9.8–25.05 %) across 8 studies (Table 3, Supplementary Table 5).

### EV-D68 coinfection

The occurrence of coinfection with EV-D68 and other pathogens was reported in 14 studies. The virus can establish coinfections with multiple viral, bacterial, and fungal pathogenic entities. The authors estimated that the rate of coinfections of EV-D68 with other pathogens was 26.79 % (95 % CI 11.89–44.49 %). The most commonly identified respiratory virus coinfected with EV-D68 was respiratory syncytial virus (23.67 %), followed by adenovirus (11.44 %) and influenza A virus (10.39 %) (Table 4, Supplementary Table 6).

**Table 4:** Coinfections of children with EV-D68 analyzed by meta-analysis.

**Variable**	**N Studies**	**n**	**prevalence**	**95% CI**	**Heterogeneity tests**			
					***Q***	***I^2^***	***t^2^***	***p***
**Coinfection (%)**	14	89	26.79	11.89–44.49	107.69	87.93	0.36	<0.001
Respiratory syncytial virus	7	40	23.67	7.01–45.13	34.41	82.57	0.26	<0.001
Rhinovirus	5	16	10.26	1.32–24.15	14.84	73.04	0.1	0.005
*Mycoplasma*	5	7	8.57	2.28–17.26	2.45	0	0	0.65
Influenza A	5	12	10.39	4.5–17.83	2.47	0	0	0.65
Influenza B a	2	4	5.13	0.67–12.3	NE	NE	NE	NE
Adenovirus a	2	7	11.44	4.1–21.26	NE	NE	NE	NE
Parainfluenza virus	3	4	4.3	0–16.45	4.73	57.68	0.06	0.09
Metapneumovirus a	2	2	0.54	0–4.59	NE	NE	NE	NE
Bocavirus	3	3	2.83	0–9.56	1.83	0	0	0.4
*Streptococcus pneumoniae*^a^	2	3	10.75	0.62–27.32	NE	NE	NE	NE
Cytomegalovirus a	2	5	3.57	0.09–9.85	NE	NE	NE	NE

*Q,* Cochran’s Q statistic for heterogeneity*; I*^2^*, I*^2^ index for the degree of heterogeneity*; t*^2^*,* tau-squared measure of heterogeneity; NE, not estimable.

a Data from two or fewer studies.

### Publication bias

Publication bias was assessed using Begg's test, which indicated significant asymmetry (*p* = 0.002) (Supplementary Fig. 2). Visual inspection of the funnel plot corroborated this finding, suggesting potential publication bias or small-study effects (Supplementary Fig. 3). Egger’s linear regression test also indicated potential bias (*p* = 0.002), consistent with Begg’s test.

## Discussion

A recent seroepidemiological systematic review on EV-D68 indicated that the seroprevalence increased quickly with age, reaching approximately 100 % by the age of 20 years, with no sign of decline throughout adulthood; this suggests continuous or frequent exposure of the populations to the virus [30]. However, the seroprevalence reflects only past infection; EV-D68 causes mild or asymptomatic infections, and cross-reactivity with antibodies against other viruses is possible. Through a systematic review and meta-analysis, the present study provides the first comprehensive evaluation of the epidemiological and clinical features of EV-D68 in respiratory specimens from Asian children.

The detection rate of EV-D68 ranged from 0.23 % to 10.56 % in the included studies, with a pooled prevalence (random-effects model) of 1.29 % (95 % CI 0.88–1.78 %). Significant heterogeneity was observed across studies (*I²* = 94.20 %), indicating that this estimate was a contextual summary. The meta-analysis confirms that EV-D68 detection in Asian children is highly heterogeneous. Many factors can influence the increase in detection rates. Previous studies have shown that the detection rate is related to children's age, national income level, geographical location, testing time, testing methods, and other factors [6]. The selection of specific subgroups was guided by factors that may significantly influence detection rates based on existing epidemiological knowledge of EV-D68. Subgroup analyses revealed national disparities, with Iran and Myanmar demonstrating markedly higher detection rates than China and other countries did (*p* < 0.001). However, the elevated rates in Iran and Myanmar derive from single-study estimates requiring cautious interpretation, potentially reflecting localized outbreaks or variations in assay sensitivity. Notably, studies conducted in high-income countries and after 2014 presented increased detection rates, which were correlated with enhanced molecular diagnostics and surveillance following North American outbreak awareness [31,32].

EV-D68 infections suggest circulation with a biennial epidemic cycle of EV-D68 infections in Europe and North America (2014, 2016, 2018), [33,34] less is known in Asia. The present study revealed irregular epidemic cycles of EV-D68 in Asia, potentially occurring every 2–4 years, which aligns with the global trends of biennial outbreaks reported in Europe and North America. The annual epidemic situation is partly affected by changes in the type of virus strains or the genetic variation of the enterovirus pathogen. However, caution is warranted in interpreting temporal trends given the sparse annual data and limited samples in the study, especially for zero-detection years.

The present analysis demonstrated increased EV-D68 susceptibility in children aged < 5 years. Consistent findings have been reported in another global review, which reported a higher detection rate of infections among children younger than 5 years of age than among those aged up to 18 years worldwide [6]. The vulnerability of children younger than 5 years of age to EV-D68 infections has been previously reported in several studies. Andres et al. [35]. reviewed EV-D68-associated respiratory cases at a hospital in Spain from 2014 to 2021 and reported that EV-D68 was mostly detected in pediatric populations (median age: 3 years), especially in patients aged < 5 years. A multicenter study of the European Nonpoliovirus Enterovirus Network (ENPEN) [36] reported that 58 institutes from 19 European countries reported 1004 EV-D68-positive samples, and that 78.9 % of infections were reported in children (ages 0–5 years). EV-D68 infections have mostly been reported in children, which is likely due to the immaturity of the immune system in children rather than in adults. The susceptibility of children to infection is also likely due to their lack of specific and cross-reactive immunity to enteroviruses [37, 38, 39]. However, the current literature demonstrates limited age-stratified reporting, with most studies predominantly reporting aggregate data — a methodological limitation potentially resulting in underestimation of the true disease burden in preschool-aged populations.

The authors found that the most common comorbidities were asthma or recurrent wheezing. However, wheezing was observed in 53 % (95 % CI 40.80–65.21 %) of the patients. Compared with other EV/RV patients, children with EV-D68 infections are more likely to have a history of asthma or recurrent wheezing [40]. A nationwide retrospective survey of hospitalizations for asthma among children was performed from January 2010 through October 2015 in Japan. The results revealed that the considerable increase in pediatric asthma hospitalizations in Japan in September 2015 was associated with the EV-D68 epidemic [41]. Using data from three surveillance systems in the United States, the analysis revealed an increase in medically attended acute respiratory illness and asthma/reactive airway disease exacerbations in children and adolescents during the summer of 2022. This increase may have been partially attributable to increased EV/RV circulation, specifically regarding the circulation of EV-D68 [42]. These results suggest that a history of recurrent wheezing or asthma is a risk factor for the detection of EV-D68 and that virus-induced wheezing/asthma might be a clinical feature of EV-D68 infection.

To explore the mechanism underlying the relationship between EV-D68 infection and asthma-like symptoms, Rajput et al. [43] developed a mouse model of EV-D68 infection and determined the mechanisms underlying airway disease. The results revealed that, in naive mice, neutrophilic inflammation and airway responsiveness were significantly greater after EV-D68 infection than after RV-A infection, which was dependent on IL-17. EV-D68 infection induces more IL-17-dependent airway inflammation and hyperresponsiveness, which is greater than that caused by RV-A infection, which is consistent with the clinical picture of severe asthma-like symptoms. Essaidi-Laziosi et al. [44] reported that global disruption of epithelial cell barrier function in patients with asthma is likely a key factor underlying increased permissiveness and susceptibility to EV-D68 infection. Currently, the specific pathogenic relationship between EV-D68 infection and asthma-like symptoms is still unclear and requires further investigation.

The present findings revealed pneumonia as the predominant manifestation of EV-D68 infection, indicating its predilection for lower respiratory tract infections (LRTIs) in pediatric populations. While 11.55 % (95 % CI 1.49–26.86 %) of cases required ICU admission, notable disparities emerged when global data were compared: a U.S. study reported substantially higher PICU admission rates (59.1 %), [45] whereas Finnish and Spanish cohorts demonstrated comparable rates (12.5 % [46] and 11.8 %, [35] respectively). This discrepancy may reflect heterogeneity in clinical cohorts and sample sizes across studies. Furthermore, the case fatality rate remained low at 0.91 %, which aligns with systematic review estimates (0 %–4.4 %), [6] suggesting a generally favorable prognosis for EV-D68-associated respiratory disease.

The present results suggest that EV-D68 is most prevalent during the summer and autumn. According to previous reports, infections caused by enteroviruses usually peak in late summer and can peak earlier in the United States in autumn [47,48]. Surveillance data from the United States revealed increases in severe respiratory illness and acute flaccid myelitis among children and adolescents with EV-D68 infections, which occurred biennially in the United States in 2014, 2016, and 2018 and primarily in the late summer and fall seasons [42]. These increases may be partially attributable to increased EV/RV circulation, specifically with respect to the circulation of EV-D68. These findings are consistent with the current findings. Pons-Salort et al. [49] used national enterovirus surveillance data from 1983 to 2013, as well as demographic and climatic data collected during the same period, to examine the patterns and drivers of the seasonality of these infections. Using mixed-effects models, they reported that climate (especially dew point temperature and latitude), but not demography, is likely to drive the seasonal pattern of enterovirus infections. This observation partly explains the seasonality of EV-D68.

In the present study, the overall coinfection rate of EV-D68 with other pathogens was 26.79 %. The most frequently detected respiratory viruses were respiratory syncytial virus and adenovirus. A multicenter study in Europe reported coinfections in 241/969 (24.9 %) EV-D68 patients, approximately half of whom also had human rhinovirus infections, followed by adenovirus and respiratory syncytial virus infections [36]. This rate is similar to the coinfection rate reported in the present study. Schuster et al. [40] reported 4 cases of coinfection with other pathogens among 339 cases of EV-D68 infection in children, and the combined infection rate was 1.2 %. The EV-D68 coinfection rates reported in different studies vary widely; however, the overall coinfection rate is not high, which is likely due to the late-summer, early-fall seasonality of the outbreaks. These findings highlight the pathogenicity of EV-D68.

There are currently no vaccines or antivirals against EV-D68 [50]. Therefore, non-pharmacological prevention strategies must be implemented. EV-D68 is usually isolated from respiratory secretions; therefore, it can spread through sneezing, coughing, and contact with surfaces contaminated by infected patients. Personal protective equipment (PPE) helps prevent the spread of EV-D68 infections. Moreover, handwashing, the use of alcoholic solutions, and wearing gloves are effective measures for preventing the spread of infection in healthcare settings [51, 52, 53].

This meta-analysis has certain limitations. First, more than half of the included studies were conducted in China or Japan. Therefore, the results of the analysis cannot accurately reflect the detection rate and clinical characteristics of EV-D68-associated respiratory infections in all Asian children. Second, more than half of the EV-D68 infections identified in the included studies were retrospectively reported; thus, the detection rate estimates were likely highly underestimated. Third, 4 of 20 studies exclusively enrolled children younger than five years old. Given that the reported median age of children infected by EV-D68 is young, the results may be skewed. Fourth, the meta-analysis demonstrated considerable heterogeneity, and the results of the subgroup analysis could not fully explain the source of the heterogeneity. In addition, the detection rate of EV-D68 via PCR is contingent upon the predominant EV-D68 clade in circulation. The currently circulating B3 clade, which is now spreading throughout the world, remains undetectable via previously established standard PCR assays because of sequence mismatches in primer/probe-binding regions. Surveillance estimates may be biased downward in years/regions where assays/primers were not updated (e.g., clade B3). Finally, the authors included data from before and after the COVID-19 pandemic. Public health measures implemented to limit the spread of SARS-CoV-2 have also disrupted the spread of respiratory viruses and enteroviruses. Additionally, the significant publication bias observed may be attributable to small-study effects, potentially leading to an overestimation of the pooled prevalence of EV-D68.

## Conclusions

Overall, the current evidence suggests that the circulation of EV-D68 is sporadic but clinically significant. EV-D68 detection in Asian children varies markedly by region and timeframe. EV-D68 infection in children is most frequently associated with pneumonia, with a generally favorable overall prognosis. The authors recommend region-specific surveillance during peak periods to guide clinical preparedness and public health responses.

## Funding sources

This study was supported by “Zhengzhou Medical and Health Science and Technology Innovation Guidance Program” (2024YLZDJH001).

## Data availability statement

The data that support the findings of this study are available from the corresponding author.

## Conflicts of interest

The authors declare no conflicts of interest.

## Appendix Supplementary materials

Image, application 1

## Footnotes

Supplementary material associated with this article can be found in the online version at doi:10.1016/j.jped.2025.101475.

## References

[1] Holm-Hansen C.C., Midgley S.E., Fischer T.K. (2016). Global emergence of enterovirus D68: a systematic review. Lancet Infect Dis.

[2] Gilrane V.L., Zhuge J., Huang W., Nolan S.M., Dhand A., Yin C. (2020). Biennial Upsurge and Molecular Epidemiology of enterovirus D68 infection in NY, USA, 2014 to 2018. J Clin Microbiol.

[3] Biggs H.M., Nix W.A., Zhang J., Rogers S., Clara W., Jara J.H. (2020). Enterovirus D68 infection among hospitalized children with severe acute respiratory illness in El Salvador and Panama, 2012-2013. Influenza Other Respir.

[4] Howson-Wells H.C., Tsoleridis T., Zainuddin I., Tarr A.W., Irving W.L., Ball J.K. (2022). Enterovirus D68 epidemic, UK, 2018, was caused by subclades B3 and D1, predominantly in children and adults, respectively, with both subclades exhibiting extensive genetic diversity. Microb Genom.

[5] Stelzer-Braid S., Yeang M., Britton P.N., Kim K.W., Varadhan H., Andrews P.I. (2022). Circulation of enterovirus D68 (EV-D68) causing respiratory illness in New South Wales, Australia, between August 2018 and November 2019. Pathology.

[6] Fall A., Kenmoe S., Ebogo-Belobo J.T., Mbaga D.S., Bowo-Ngandji A., Foe-Essomba J.R. (2022). Global prevalence and case fatality rate of Enterovirus D68 infections, a systematic review and meta-analysis. PLoS Negl Trop Dis.

[7] Grizer C.S., Messacar K., Mattapallil J.J. (2024). Enterovirus-D68 - A reemerging non-polio Enterovirus that causes severe Respiratory and neurological disease in children. Front Virol.

[8] Page M.J., McKenzie J.E., Bossuyt P.M., Boutron I., Hoffmann T.C., Mulrow C.D. (2021). The PRISMA 2020 statement: an updated guideline for reporting systematic reviews. BMJ.

[9] Munn Z., Moola S., Riitano D., Lisy K. (2014). The development of a critical appraisal tool for use in systematic reviews addressing questions of prevalence. Int J Health N Hav Policy Manag.

[10] Imamura T., Fuji N., Suzuki A., Tamaki R., Saito M., Aniceto R. (2011). Enterovirus 68 among children with severe acute respiratory infection, the Philippines. Emerg Infect Dis.

[11] Kaida A., Kubo H., Sekiguchi J., Kohdera U., Togawa M., Shiomi M. (2011). Enterovirus 68 in children with acute respiratory tract infections, Osaka, Japan. Emerg Infect Dis.

[12] Ikeda T., Mizuta K., Abiko C., Aoki Y., Itagaki T., Katsushima F. (2012). Acute respiratory infections due to enterovirus 68 in Yamagata, Japan between 2005 and 2010. Microbiol Immunol.

[13] Linsuwanon P., Puenpa J., Suwannakarn K., Auksornkitti V., Vichiwattana P., Korkong S. (2012). Molecular epidemiology and evolution of human enterovirus serotype 68 in Thailand, 2006-2011. PLoS One.

[14] Imamura T., Suzuki A., Lupisan S., Okamoto M., Aniceto R., Egos R.J. (2013). Molecular evolution of enterovirus 68 detected in the Philippines. PLoS One.

[15] Lu Q.B., Wo Y., Wang H.Y., Wei M.T., Zhang L., Yang H. (2013). Detection of enterovirus 68 as one of the commonest types of enterovirus found in patients with acute respiratory tract infection in China. J Med Microbiol.

[16] Furuse Y., Chaimongkol N., Okamoto M., Imamura T., Saito M., Tamaki R. (2015). Molecular epidemiology of enterovirus D68 from 2013 to 2014 in Philippines. J Clin Microbiol.

[17] Xiao Q., Ren L., Zheng S., Wang L., Xie X., Deng Y. (2015). Prevalence and molecular characterizations of enterovirus D68 among children with acute respiratory infection in China between 2012 and 2014. Sci Rep.

[18] Kaida A., Iritani N., Yamamoto S., Kanbayashi D., Hirai Y., Kohdera U. (2016). Single genetic clades of EV-D68 strains in 2010, 2013, and 2015 in Osaka City. Japan. J Clin Virol..

[19] Lam H.Y., Wong A.T., Tsao Y.C., Tang B.S. (2016). Prevalence and phylogenetic characterization of human enterovirus D68 among children with respiratory infection in Hong Kong. Diagn Micr Infec Dis.

[20] Thongpan I., Wanlapakorn N., Vongpunsawad S., Linsuwanon P., Theamboonlers A., Payungporn S. (2015). Prevalence and phylogenetic characterization of enterovirus D68 in pediatric patients with acute Respiratory tract infection in Thailand. Jpn J Infect Dis.

[21] Zhang T., Li A., Chen M., Wu J., Huang F. (2016). Respiratory infections associated with enterovirus D68 from 2011 to 2015 in Beijing. China. J Med Virol..

[22] Itagaki T., Aoki Y., Matoba Y., Tanaka S., Ikeda T., Mizuta K. (2017). Clinical characteristics of children infected with enterovirus D68 in an outpatient clinic and the association with bronchial asthma. Infect Dis.

[23] Shen L., Gong C., Xiang Z., Zhang T., Li M., Li A. (2019). Upsurge of enterovirus D68 and circulation of the new subclade D3 and subclade B3 in Beijing, China, 2016. Sci Rep.

[24] Hasuwa T., Kinoshita F., Harada S., Nakashima K., Yoshihara K., Toku Y. (2020). Viral etiology of acute lower Respiratory tract infections in hospitalized children in Nagasaki, a regional City of Japan in 2013-2015. Pediatr Infect Dis J.

[25] Mozhgani S.H., Keshavarz M., Mousavi N., Namdari H., Salimi V., Mokhtari-Azad T. (2020). Frequent detection of enterovirus D68 and rhinovirus type C in children with acute respiratory infections. Eur J Clin Microbiol.

[26] Tang S.H., Yuan Y., Xie Z.H., Chen M.J., Fan X.D., Guo Y.H. (2021). Enterovirus D68 in hospitalized children with respiratory symptoms in Guangdong from 2014 to 2018: molecular epidemiology and clinical characteristics. J Clin Virol.

[27] Li Q., Chen X., Ai J., Li L., Li C., Zhu Y. (2023). Clinical and molecular epidemiologic features of enterovirus D68 infection in children with acute lower respiratory tract infection in China. Arch Virol.

[28] Ikuse T., Aizawa Y., Kachikawa R., Kamata K., Osada H., Win S.M.K. (2024). Detection of enterovirus D68 among children with severe acute respiratory infection in Myanmar. J Microbiol Immunol.

[29] Li F., Lu R.J., Zhang Y.H., Shi P., Ao Y.Y., Cao L.F. (2024). Clinical and molecular epidemiology of enterovirus D68 from 2013 to 2020 in Shanghai. Sci Rep.

[30] Jorgensen D., Grassly N.C. (2025). Pons-Salort M. Global age-stratified seroprevalence of enterovirus D68: a systematic literature review. Lancet Microbe.

[31] Midgley S.E., Benschop K., Dyrdak R., Mirand A., Bailly J.L., Bierbaum S. (2020). Co-circulation of multiple enterovirus D68 subclades, including a novel B3 cluster, across Europe in a season of expected low prevalence, 2019/20. Euro Surveill.

[32] Xiang Z., Wang J. (2016). Enterovirus D68 and Human Respiratory infections. Semin Respir Crit Care.

[33] Kramer R., Sabatier M., Wirth T., Pichon M., Lina B., Schuffenecker I. (2018). Molecular diversity and biennial circulation of enterovirus D68: a systematic screening study in Lyon, France, 2010 to 2016. Eurosurveillance.

[34] Messacar K., Pretty K., Reno S., Dominguez S.R. (2019). Continued biennial circulation of enterovirus D68 in CO. J Clin Virol.

[35] Andrés C., Vila J., Creus-Costa A., Piñana M., González-Sánchez A., Esperalba J. (2022). Enterovirus D68 in hospitalized children, Barcelona, Spain, 2014-2021. Emerg Infect Dis.

[36] Simoes M.P., Hodcroft E.B., Simmonds P., Albert J., Alidjinou E.K., Ambert-Balay K. (2024). Epidemiological and clinical insights into the enterovirus D68 upsurge in Europe 2021/22 and the emergence of novel B3-derived lineages, ENPEN multicentre study. J Infect Dis.

[37] Renois F., Bouin A., Andreoletti L. (2012). Enterovirus 68 in pediatric patients hospitalized for acute airway diseases. J Clin Microbiol.

[38] Khan F. (2015). Enterovirus D68: acute respiratory illness and the 2014 outbreak. Emerg Med Clin North Am.

[39] Meijer A., van der Sanden S., Snijders B.E., Jaramillo-Gutierrez G., Bont L., van der Ent C.K. (2011). Emergence and epidemic occurrence of enterovirus 68 respiratory infections in The Netherlands in 2010. Virology.

[40] Schuster J.E., Selvarangan R., Hassan F., Briggs K.B., Hays L., Miller J.O. (2017). Clinical course of enterovirus D68 in hospitalized children. Pediatr Infect Dis J.

[41] Korematsu S., Nagashima K., Sato Y., Nagao M., Hasegawa S., Nakamura H. (2017). “Spike” in acute asthma exacerbations during entervirus D68 epidemic in Japan: a nation-wide survey. Allergol Int.

[42] Ma K.C., Winn A., Moline H.L., Scobie H.M., Midgley C.M., Kirking H.L. (2022). Increase in acute Respiratory illnesses among children and adolescents associated with rhinoviruses and enteroviruses, including enterovirus D68 - United States, July-September 2022. MMWR-Morbid Mortal W.

[43] Rajput C., Han M., Bentley J.K., Lei J., Ishikawa T., Wu Q. (2018). Enterovirus D68 infection induces IL-17-dependent neutrophilic airway inflammation and hyperresponsiveness. JCI Insight.

[44] Essaidi-Laziosi M., Royston L., Boda B., Pérez-Rodriguez F.J., Piuz I., Hulo N. (2023). Altered cell function and increased replication of rhinoviruses and EV-D68 in airway epithelia of asthma patients. Front Microbiol.

[45] Cao R.G., Mejias A., Leber A.L., Wang H. (2023). Clinical and molecular characteristics of the 2022 Enterovirus-D68 outbreak among hospitalized children, OH, USA. J Clin Virol.

[46] Peltola V., Österback R., Waris M., Ivaska L., Tähtinen P.A., Laine M. (2023). Enterovirus D68 outbreak in children, Finland, August-September 2022. Emerg Infect Dis.

[47] Abedi G.R., Watson J.T., Pham H., Nix W.A., Oberstr M.S., Gerber S.I. (2015). Enterovirus Surveillance - United States, 2009-2013. MMWR-Morbid Mortal W.

[48] Abedi G.R., Watson J.T., Nix W.A., Oberstr M.S., Gerber S.I. (2018). Enterovirus and Human parechovirus surveillance-United States, 2014-2016. MMWR-Morbid Mortal W.

[49] Pons-Salort M., Oberste M.S., Pallansch M.A., Abedi G.R., Takahashi S., Grenfell B.T. (2018). The seasonality of nonpolio enteroviruses in the United States: patterns and drivers. Proc Natl Acad Sci USA.

[50] Hu Y., Musharrafieh R., Zheng M., Wang J. (2020). Enterovirus D68 antivirals: past, present, and future. ACS Infect Dis.

[51] Lugo D., Krogstad P. (2016). Enteroviruses in the early 21st century: new manifestations and challenges. Curr Opin Pediatr.

[52] Principi N., Esposito S. (2015). Enterovirus D-68: an emerging cause of infection. Expert Rev Respir Med.

[53] Murray J.S., Mirch M.C., Amin P.M. (2015). Enterovirus D68: what pediatric healthcare professionals need to know. J Spec Pediatr Nurs.

