# Supplementary material for: Epidemiological and clinical characteristics associated with enterovirus D68 respiratory diseases in Asian children: a systematic review and meta-analysis
Source: J Pediatr (Rio J). 2025 Nov 14;102(1):101475. doi: 10.1016/j.jped.2025.101475 (PMC12663508; doi:10.1016/j.jped.2025.101475)

**JPED-D-25-00247_ Supplementary Material**

**Supplementary Table 1** The Search strategy of EV-D68.

| Databases | Searches |
| --- | --- |
| PubMed | ("Enterovirus D, Human"[Mesh]) OR ("enterovirus D68"[Title/Abstract] OR "enterovirus-D68"[Title/Abstract] OR "enterovirus-68"[Title/Abstract] OR "enterovirus 68"[Title/Abstract] OR "EV-68"[Title/Abstract] OR "EV-D68"[Title/Abstract] OR "EV D68"[Title/Abstract] OR "D68"[Title/Abstract] OR "HEV-D68"[Title/Abstract] OR "hev-68"[Title/Abstract] OR "hev 68"[Title/Abstract] OR "ev 68"[Title/Abstract]) |
| Embase | enterovirus d68'/exp OR 'enterovirus 68':ab,kw,ti OR 'ev-d68':ab,kw,ti OR 'enterovirus-d68':ab,kw,ti OR 'enterovirus-68':ab,kw,ti OR 'ev-68':ab,kw,ti OR 'ev d68':ab,kw,ti OR 'd68':ab,kw,ti OR 'hev-d68':ab,kw,ti OR 'hev-68':ab,kw,ti OR 'hev 68':ab,kw,ti OR 'ev68':ab,kw,ti |
| Web of Science | (((((((((((TS=("enterovirus D68")) OR TS=(enterovirus-D68)) OR TS=(enterovirus-68)) OR TS=("enterovirus 68")) OR TS=(EV-68)) OR TS=(EV-D68)) OR TS=("EV D68")) OR TS=(D68)) OR TS=(HEV-D68)) OR TS=(HEV-68)) OR TS=("EV 68")) OR TS=("HEV 68") |
| Scopus | TITLE-ABS-KEY(enterovirus D68) OR TITLE-ABS-KEY(enterovirus-D68) OR TITLE-ABS-KEY(enterovirus-68) OR TITLE-ABS-KEY("enterovirus 68") OR TITLE-ABS-KEY(EV-68) OR TITLE-ABS-KEY(EV-D68) OR TITLE-ABS-KEY("EV D68") OR TITLE-ABS-KEY(D68) OR TITLE-ABS-KEY(HEV-D68) OR TITLE-ABS-KEY(HEV-68) OR TITLE-ABS-KEY("EV 68") OR TITLE-ABS-KEY("HEV 68") |
| WanFang database | 主题:("肠道病毒 D68") or 主题:(肠道病毒-D68) or 主题:(肠道病毒D组68型) or 主题:("肠道病毒 68") or主题:("肠病毒 68") |
| VIP database | (M="肠道病毒 D68" OR M="肠道病毒-D68" OR M="肠道病毒D组68型" OR M="肠道病毒 68" OR M="肠病毒 68" ) |
| China National Knowledge Infrastructure (CNKI) | (TKA="肠道病毒D68" OR TKA="肠道病毒-D68" OR TKA="肠道病毒D组68型" OR TKA="肠道病毒68" OR TKA="肠病毒68" ) |
| SinoMed | 肠道病毒D68[常用字段:智能] OR "肠道病毒-D68"[常用字段:智能] OR "肠道病毒D组68型"[常用字段:智能] OR "肠道病毒68"[常用字段:智能] OR "肠病毒68"[常用字段:智能] |

**Supplementary Table** 2 **Characteristics and summary of outcomes from the 20 included studies.**

| **First author** | **Year** | **Timing of samples collection** | **Country** | **Country income level** | **Study period** | **Sample type** | **Diagnostic method** | **Age range** |
| --- | --- | --- | --- | --- | --- | --- | --- | --- |
| Imamura^10^ | 2011 | Retrospectively | Philippines | Lower-middle-income economies | mid-May 2008 - mid-May 2009 | NS | Classical RT-PCR | 7 days -14 years |
| Kaida^11^ | 2011 | Prospectively | Japan | High-income economies | October 2009 - October 2010 | NM, SPU, TS | Classical RT-PCR | 1 month - 5 years |
| Ikeda^12^ | 2012 | Prospectively | Japan | High-income economies | 2005 - 2010 | NS | Classical RT-PCR | Unclear |
| Linsuwanon^13^ | 2012 | Retrospectively | Thailand | Upper-middle-income economies | February 2006 - November 2011 | NPA, NS | Classical RT-PCR | 0 -18 years |
| Imamura14 | 2013 | Prospectively | Philippines | Lower-middle-income economies | June 2009 - November 2011 | NS | Classical RT-PCR | 4 days -14 years |
| Lu15 | 2014 | Retrospectively | China | Upper-middle-income economies | September 2009 - June 2012 | NS | Real -Time RT-PCR | 1 month -14 years |
| Furuse16 | 2015 | Retrospectively | Philippines | Lower-middle-income economies | September 2012 - February 2014 | NS | Classical RT-PCR | Unclear |
| Xiao17 | 2015 | Prospectively | China | Upper-middle-income economies | January 2012 - November 2014 | NS | Classical RT-PCR | 1 month -16 years |
| Kaida18 | 2016 | Prospectively | Japan | High-income economies | November 2010 - December 2015 | Respiratory samples | Real -Time RT-PCR | 0 - 10 years |
| Thongpan^20^ | 2016 | Retrospectively | Thailand | Upper-middle-income economies | 2012 - 2014 | NS, TS | Classical RT-PCR | 0 - 5 years |
| Zhang^21^ | 2016 | Retrospectively | China | Upper-middle-income economies | January 2011 - July 2015 | NS, SPU | Real -Time RT-PCR | 0 - 14 years |
| Lam19 | 2016 | Retrospectively | China | Upper-middle-income economies | January - December 2014 | NS, TS, NPA | Real -Time RT-PCR | 0 - 18 years |
| Itagaki22 | 2017 | Retrospectively | Japan | High-income economies | January 2010 - December 2015 | NS | Classical RT-PCR | 0 -16 years |
| Shen23 | 2019 | Retrospectively | China | Upper-middle-income economies | 2015 - 2017 | NS, SPU | Classical RT-PCR | 0 - 18 years |
| Hasuwa24 | 2020 | Prospectively | Japan | High-income economies | February 2013 - January 2015 | NS | Classical RT-PCR | 0 - 5 years |
| Mozhgani25 | 2021 | Prospectively | Iran | Upper-middle-income economies | January - December 2018 | NS | Real -Time RT-PCR | 0 - 5 years |
| Tang26 | 2021 | Retrospectively | China | Upper-middle-income economies | January 2014 - July 2018 | NS | Real -Time RT-PCR | 0 -14 years |
| Li27 | 2023 | Prospectively | China | Upper-middle-income economies | May 2017 - December 2019 | NS, SPU | Real -Time RT-PCR | Unclear |
| Ikuse28 | 2024 | Retrospectively | Myanmar | Lower-middle-income economies | May 2017 - January 2019 | NS | Classical RT-PCR | 0 - 12 years |
| Li29 | 2024 | Retrospectively | China | Upper-middle-income economies | June 2013 - December 2020 | NS, BAL | Real -Time RT-PCR | 0 - 18 years |

NS, nasopharyngeal swab; BAL, bronchoalveolar lavage; SPU, sputum; NM, Nasal mucus; TS, Throat swab；NPA, Nasopharyngeal aspiration specimens.

**Supplementary Table 2** Characteristics and summary of outcomes from the 20 included studies.

| **First author** | **Year** | **Sample size** | **Median age (y)** | **Male（%）** |
| --- | --- | --- | --- | --- |
| Imamura10 | 2011 | 816 | 0.75 | 432（52.9） |
| Kaida11 | 2011 | 448 | 3.45 | 258（57.6） |
| Ikeda12 | 2012 | 6307 | - | - |
| Linsuwanon13 | 2012 | 1810 | - | - |
| Imamura^14^ | 2013 | 1187 | 0.92 | - |
| Lu^15^ | 2014 | 1565 | 0.75 | 1034（66.1） |
| Furuse^16^ | 2015 | 1854 | - | - |
| Xiao^17^ | 2015 | 1876 | 0.75 | 1238（66.0） |
| Kaida^18^ | 2016 | 2215 | - | - |
| Thongpan20 | 2016 | 837 | - | - |
| Zhang21 | 2016 | 3457 | - | - |
| Lam^19^ | 2016 | 1461 | - | - |
| Itagaki^22^ | 2017 | 5794 | - | - |
| Shen^23^ | 2019 | 6988 | - | - |
| Hasuwa^24^ | 2020 | 373 | 1.25 | - |
| Mozhgani^25^ | 2021 | 322 | - | 170（52.8） |
| Tang^26^ | 2021 | 2503 | 1.4 | 1635（65.3） |
| Li^27^ | 2023 | 3071 | - | - |
| Ikuse^28^ | 2024 | 570 | - | 321（56.3） |
| Li^29^ | 2024 | 3997 | - | - |

**Supplemental Table 3** Qualities of studies included in the systematic review and meta-analysis.

| First author | Publication year | Q1 | Q2 | Q3 | Q4 | Q5 | Q6 | Q7 | Q8 | Q9 | Total |
| --- | --- | --- | --- | --- | --- | --- | --- | --- | --- | --- | --- |
| Imamura10 | 2011 | Y | Y | Y | Y | Y | Y | Y | Y | Y | 9 |
| Kaida11 | 2011 | Y | Y | Y | Y | Y | N | Y | Y | U | 7 |
| Ikeda12 | 2012 | Y | Y | Y | Y | Y | Y | Y | Y | U | 8 |
| Linsuwanon13 | 2012 | Y | Y | Y | Y | Y | Y | Y | Y | Y | 9 |
| Imamura^14^ | 2013 | Y | Y | Y | Y | Y | N | Y | Y | U | 7 |
| Lu^15^ | 2014 | Y | Y | Y | N | Y | N | Y | Y | U | 6 |
| Furuse^16^ | 2015 | Y | Y | Y | Y | Y | Y | Y | Y | U | 8 |
| Xiao^17^ | 2015 | Y | Y | Y | Y | Y | Y | Y | Y | Y | 9 |
| Kaida^18^ | 2016 | Y | Y | Y | N | Y | Y | Y | Y | U | 7 |
| Thongpan20 | 2016 | Y | Y | Y | N | Y | Y | Y | Y | U | 7 |
| Zhang21 | 2016 | Y | Y | Y | N | Y | N | Y | Y | Y | 7 |
| Lam19 | 2016 | Y | Y | Y | N | Y | N | Y | Y | Y | 7 |
| Itagaki22 | 2017 | Y | Y | Y | Y | Y | Y | Y | Y | Y | 9 |
| Shen^23^ | 2019 | Y | Y | Y | Y | Y | Y | Y | Y | Y | 9 |
| Hasuwa24 | 2020 | Y | Y | Y | Y | Y | N | Y | Y | U | 7 |
| Mozhgani25 | 2021 | Y | Y | N | Y | Y | N | Y | Y | U | 6 |
| Tang26 | 2021 | Y | Y | Y | Y | Y | Y | Y | Y | Y | 9 |
| Li27 | 2023 | Y | Y | Y | Y | Y | Y | Y | Y | Y | 9 |
| Ikuse28 | 2024 | Y | Y | Y | Y | Y | Y | Y | Y | U | 8 |
| Li29 | 2024 | Y | Y | Y | Y | Y | Y | Y | Y | Y | 9 |

Notes:

Q1–Q9 represents questions used to assess the quality of included studies, which are listed below

Q1. Was the sample frame appropriate to address the target populations? Q2. Were the study participants sampled appropriately? Q3. Was the sample size adequate? Q4. Were the study subjects and setting described in detail? Q5. Was the data analysis conducted with sufficient coverage of the identified sample? Q6. Was a valid method used in the identification of conditions? Q7. Was the condition measured in a standard, reliable way for all participants? Q8. Was there an appropriate statistical analysis? Q9. Was the response rate adequate, and if not, was the low response rate managed appropriately?

**Supplementary Table** **4** The demographics and comorbidities of children with EV-D68 analyzed by meta-analysis.

| **ID** | **Study** | **N** | **Median age(IQR）or Mean age (range)** | **n(%)** | | | |
| --- | --- | --- | --- | --- | --- | --- | --- |
|  |  |  |  | **<5 y** | **6-14 y** | **15-18 y** | **Male** |
| 1 | Imamura(2011)^10^ | 21 | 1.75 (0.08-9) | - | - | - | 13（61.9） |
| 2 | Kaida(2011)^11^ | 15 | 2.83 (IQR:1.25-4.08) | 15（100） | - | - | 10（66.67） |
| 3 | Ikeda(2012)^12^ | 55 | 5.2 (IQR:0.42-15） | 31（56.36） | 14（25.45） | - | 33（60） |
| 4 | Linsuwanon(2012)^13^ | 25 | 7.6±5 ^a^ | 9（36） | 14（56） | 2（8） | 10（40） |
| 5 | Imamura(2013)^14^ | 9 | - | - | - | - | - |
| 6 | Lu(2014)^15^ | 7 | 3(IQR:2-10) | 5（71.43） | 2（28.57） | - | 6（85.71） |
| 7 | Furuse(2015)^16^ | 20 | 1.17(0.08-4) | - | - | - | - |
| 8 | Xiao(2015)^17^ | 19 | 2.67(0.08-10.67) | 17（89.47） | 2（10.53） | - | 10（52.63） |
| 9 | Kaida(2016)^18^ | 18 | 1.58（IQR:3.92-0.83） | 16（88.89） | 2（11.11） | - | 9（50） |
| 10 | Thongpan(2016)^20^ | 5 | 3（IQR:1.5-4) | 5（100） | - | - | 3（60） |
| 11 | Zhang(2016)^21^ | 11 | 8(IQR:5-10) | 3（27.27） | 7（63.64） | 1（9.09） | 6（54.55） |
| 12 | Lam(2016)^19^ | 24 | 4(IQR:7-10) | 15（62.5） | 9（37.5） | - | 16（66.67） |
| 13 | Itagaki(2017)^22^ | 79 | 3.5y(IQR:0.42-15) | - | - | - | 36（45.57） |
| 14 | Shen(2019)^23^ | 20 | 5（IQR:1.5-8.5） | 13（65） | 7（35） | - | 15（75） |
| 15 | Hasuwa(2020)^24^ | 10 | 2.75（IQR:2.42-4.25） | - | - | - | - |
| 16 | Mozhgani(2020)^25^ | 34 | - | 34（100） | - | - | 19（55.88） |
| 17 | Tang(2021)^26^ | 17 | 1.83(IQR:0.23-4.69) | 15（88.24） | 2（11.76） | - | 11（64.71） |
| 18 | Li(2023)^27^ | 10 | - | 7（70） | 3（30） | - | 9（90） |
| 19 | Ikuse(2024)^28^ | 42 | 8.5(IQR:4-12) | - | - | - | 18（42.86） |
| 20 | Li(2024)^29^ | 9 | 1.3(IQR:0.4-5.2) | 9（100） | - | - | 7（77.78） |

**^a^ Represents the mean±Standard deviation.**

**Supplementary Table** **4** The demographics and comorbidities of children with EV-D68 analyzed by meta-analysis(continued).

| **ID** | **Study** | **N** | **n(%)** | | | | | |
| --- | --- | --- | --- | --- | --- | --- | --- | --- |
|  |  |  | **Comorbidities** | **asthma or recurrent wheezing** | **Cardiovascular disease** | **Immunosuppression** | **Gastrointestinal disease** | **Neurological disease** |
| 1 | Imamura(2011)^10^ | 21 | - | - | - | - | - | - |
| 2 | Kaida(2011)^11^ | 15 | - | - | - | - | - | - |
| 3 | Ikeda(2012)^12^ | 55 | - | - | - | - | - | - |
| 4 | Linsuwanon(2012)^13^ | 25 | 4(16) | 2(8) | 1(4) | - | 1(4) | - |
| 5 | Imamura(2013)^14^ | 9 | - | - | - | - | - | - |
| 6 | Lu(2014)^15^ | 7 | - | - | - | - | - | - |
| 7 | Furuse(2015)^16^ | 20 | - | - | - | - | - | - |
| 8 | Xiao(2015)^17^ | 19 | 13(68.42) | 13(68.42) | - | - | - | - |
| 9 | Kaida(2016)^18^ | 18 | - | - | - | - | - | - |
| 10 | Thongpan(2016)^20^ | 5 | 2(40) | 2(40) | - | - | - | - |
| 11 | Zhang(2016)^21^ | 11 | - | - | - | - | - | - |
| 12 | Lam(2016)^19^ | 24 | - | - | - | - | - | - |
| 13 | Itagaki(2017)^22^ | 79 | 29(37.71) | 29(37.71) | - | - | - | - |
| 14 | Shen(2019)^23^ | 20 | 0(0) | 0(0) | - | - | - | - |
| 15 | Hasuwa(2020)^24^ | 10 | 5(50) | 5(50) | - | - | - | - |
| 16 | Mozhgani(2020)^25^ | 34 | - | - | - | - | - | - |
| 17 | Tang(2021)^26^ | 17 | 3(17.65) | - | - | - | - | 3（17.64） |
| 18 | Li(2023)^27^ | 10 | 0(0) | 0(0) | - | - | - | - |
| 19 | Ikuse(2024)^28^ | 42 | - | - | - | - | - | - |
| 20 | Li(2024)^29^ | 9 | 4(44.44) | 1(11.11) | 2(22.22) | 1(11.11) | - | - |

**Supplementary Table 5** The clinical characteristics and seasonality of children with EV-D68 analyzed by meta-analysis.

| **ID** | **Study** | **N** | **n (%)** | | | | | | | | | |
| --- | --- | --- | --- | --- | --- | --- | --- | --- | --- | --- | --- | --- |
|  |  |  | **URTI** | **pneumonia** | **asthmatic bronchitis** | **bronchial asthma** | **Bronchiolitis** | **bronchitis** | **Fever** | **cough** | **wheezing** | **sputum production** |
| 1 | Imamura(2011)^10^ | 21 | - | 21（100） | - | - | - | - | - | 21（100） | 14（66.67） | - |
| 2 | Kaida(2011)^11^ | 15 | 2（13.33） | 5（33.33） | 6（40） | 1（6.67） | - | 1（6.67） | 10（66.67） | 1（6.67） | 9（60） | - |
| 3 | Ikeda(2012)^12^ | 55 | 41（74.55） | - | 3（5.45） | 6（10.91） | - | 5（9.09） | - | - | 11（20） | - |
| 4 | Linsuwanon(2012)^13^ | 25 | 16（64） | 8（32） | - | 1（4） | - | - | 23（92） | 8（32） | 7（28） | - |
| 5 | Imamura(2013)^14^ | 9 | - | 9（100） | - | - | - | - | - | 9（100） | 5（55.56） | - |
| 6 | Lu(2014)^15^ | 7 | - | 7（100） | - | 3（42.86） | - | - | 7（100） | 7（100） | 4（57.14） | 5（71.43） |
| 7 | Furuse(2015)^16^ | 20 | - | - | - | - | - | - | - | - | 7（35） | - |
| 8 | Xiao(2015)^17^ | 19 | - | 14（73.68） | - | 12（63.16） | 1（5.26） | 1（5.26） | 5（26.32） | 19（100） | 17（89.47） | 5（26.32） |
| 9 | Kaida(2016)^18^ | 18 | - | 6（33.33） | 5（27.78） | 4（22.22） | - | 3（16.67） | - | - | 9（50） | - |
| 10 | Thongpan(2016)^20^ | 5 | - | 5（100） | - | - | - | - | 4（80） | 5（100） | 5（100） | - |
| 11 | Zhang(2016)^21^ | 11 | - | - | - | - | - | - | 8（72.73） | 6（54.55） | - | 4（36.36） |
| 12 | Lam(2016)^19^ | 24 | 16（66.67） | 7（29.17） | 17（70.83） | - | - | - | 21（87.5） | - | 17（70.83） | - |
| 13 | Itagaki(2017)^22^ | 79 | 44（55.7） | - | - | 14（17.72） | 10（12.66） | 7（8.86） | 48（60.76） | 66（83.54） | 24（30.38） | - |
| 14 | Shen(2019)^23^ | 20 | 5（25） | 15（75） | - | - | - | - | 11（55） | 19（95） | - | 11（55） |
| 15 | Hasuwa(2020)^24^ | 10 | - | 6（60） | - | - | - | - | - | - | - | - |
| 16 | Mozhgani(2020)^25^ | 34 | - | - | - | - | - | - | - | - | - | - |
| 17 | Tang(2021)^26^ | 17 | 2（11.76） | 12（70.59） | - | - | - | - | 10（58.82） | 14（82.35） | 12（70.59） | 10（58.82） |
| 18 | Li(2023)^27^ | 10 | - | 8（80） | - | - | 2（20） | - | 0（0） | 10（100） | 5（50） | - |
| 19 | Ikuse(2024)^28^ | 42 | - | 42（100） | - | - | - | - | 19（45.24） | 42（100） | 16（38.1） | - |
| 20 | Li(2024)^29^ | 9 | - | 9（100） | - | - | - | - | 4（44.44） | 9（100） | 5（55.56） | - |

URTI, upper respiratory tract infection.

**Supplementary Table 5** The clinical characteristics and seasonality of children with EV-D68 analyzed by meta-analysis(continued).

| **ID** | **Study** | **N** | **n (%)** | | | | | | | | |
| --- | --- | --- | --- | --- | --- | --- | --- | --- | --- | --- | --- |
|  |  |  | **chest ache** | **dyspnea** | **sore throat** | **Runny nose** | **sneezing** | **vomiting** | **diarrhea** | **ICU** | **Death** |
| 1 | Imamura(2011)^10^ | 21 | - | 18（85.71） | - | - | - | - | - | - | 2（9.52%） |
| 2 | Kaida(2011)^11^ | 15 | - | 1（6.67） | 1（6.67） | - | - | - | - | 1 | - |
| 3 | Ikeda(2012)^12^ | 55 | - | - | - | - | - | - | - | - | - |
| 4 | Linsuwanon(2012)^13^ | 25 | - | 7（28） | - | - | - | - | - | 1 | 0（0%） |
| 5 | Imamura(2013)^14^ | 9 | - | 7（77.78） | - | - | - | - | - | - | 1（11.11%） |
| 6 | Lu(2014)^15^ | 7 | 1（14.29） | 3（42.86） | - | 1（14.29） | - | - | 3（42.86） | 3 | 0（0%） |
| 7 | Furuse(2015)^16^ | 20 | - | - | - | - | - | - | - | - | 1（5%） |
| 8 | Xiao(2015)^17^ | 19 | 2（10.53） | - | - | 3（15.79） | - | - | 4（21.05） | 5 | - |
| 9 | Kaida(2016)^18^ | 18 | - | - | - | - | - | - | - | 1 | 0（0%） |
| 10 | Thongpan(2016)^20^ | 5 | - | 3（60） | - | 4（80） | - | 2（40） | - | 1 | 0（0%） |
| 11 | Zhang(2016)^21^ | 11 | 2（18.18） | - | 2（18.18） | - | 1（9.09） | - | - | 0 | 0（0%） |
| 12 | Lam(2016)^19^ | 24 | - | - | - | - | - | - | - | 0 | 0（0%） |
| 13 | Itagaki(2017)^22^ | 79 | - | - | - | 50（63.29） | - | - | 2（2.53） | - | - |
| 14 | Shen(2019)^23^ | 20 | - | 1（5） | 6（30） | 3（15） | - | - | 1（5） | - | 0（0%） |
| 15 | Hasuwa(2020)^24^ | 10 | - | - | - | - | - | - | - | 0 | 0（0%） |
| 16 | Mozhgani(2020)^25^ | 34 | - | - | - | - | - | - | - | - | - |
| 17 | Tang(2021)^26^ | 17 | - | 5（29.41） | - | 3（17.65） | 1（5.88） | 1（5.88） | 0（0） | 15 | 0（0%） |
| 18 | Li(2023)^27^ | 10 | - | 3（30） | 0（0） | 1（10） | - | - | - | 0 | 0（0%） |
| 19 | Ikuse(2024)^28^ | 42 | - | 31（73.81） | - | 19（45.24） | - | - | - | 4 | 3（7.14%） |
| 20 | Li(2024)^29^ | 9 | - | - | - | - | - | - | 1（11.11） | - | 0（0%） |

**Supplementary Table** **5** The clinical characteristics and seasonality of children with EV-D68 analyzed by meta-analysis(continued).

| **ID** | **Study** | **N** | **n (%)** | | | |
| --- | --- | --- | --- | --- | --- | --- |
|  |  |  | **Spring** | **Summer** | **Autumn** | **Winter** |
| 1 | Imamura(2011)^10^ | 21 | - | - | 9（42.86） | 12（57.14） |
| 2 | Kaida(2011)^11^ | 15 | - | 12（80） | 3（20） | - |
| 3 | Ikeda(2012)^12^ | 55 | - | 7（12.73） | 48（87.27） | - |
| 4 | Linsuwanon(2012)^13^ | 25 | 2（8） | 13（52） | 5（20） | 5（20） |
| 5 | Imamura(2013)^14^ | 9 | - | 9（100） | - | - |
| 6 | Lu(2014)^15^ | 7 | 1（14.29） | - | 3（42.86） | 3（42.86） |
| 7 | Furuse(2015)^16^ | 20 | 4（20） | - | 2（10） | 14（70） |
| 8 | Xiao(2015)^17^ | 19 | - | - | 16（84.21） | 3（15.79） |
| 9 | Kaida(2016)^18^ | 18 | - | 5（27.78） | 12（66.67） | 1（5.56） |
| 10 | Thongpan(2016)^20^ | 5 | - | - | 3（60） | 2（40） |
| 11 | Zhang(2016)^21^ | 11 | - | 2（18.18） | 9（81.82） | - |
| 12 | Lam(2016)^19^ | 24 | 7（29.17） | 14（58.33） | 3（12.5） | - |
| 13 | Itagaki(2017)^22^ | 79 | - | 5（6.33） | 74（93.67） | - |
| 14 | Shen(2019)^23^ | 20 | - | 14（70） | 6（30） | - |
| 15 | Hasuwa(2020)^24^ | 10 | 4（40） | 5（50） | 1（10） | - |
| 16 | Mozhgani(2020)^25^ | 34 | 3（8.82） | - | 2（5.88） | 29（85.29） |
| 17 | Tang(2021)^26^ | 17 | 4（23.53） | 7（41.18） | 6（35.29） | - |
| 18 | Li(2023)^27^ | 10 | - | - | - | - |
| 19 | Ikuse(2024)^28^ | 42 | - | 12（28.57） | 29（69.05） | 1（2.38） |
| 20 | Li(2024)^29^ | 9 | 1（11.11） | 4（44.44） | 4（44.44） | - |

| **ID** | **Study** | **N** | **n (%)** | | | | | | | | | | | |
| --- | --- | --- | --- | --- | --- | --- | --- | --- | --- | --- | --- | --- | --- | --- |
|  |  |  | **Coinfection** | **RSV** | **RV** | **Flu A** | **Flu B** | **AdV** | **PIV** | **HMPV** | **BoV** | **HCoV** | **Cox B4** | **Parechovirus** |
| 1 | Imamura(2011)^10^ | 21 | - | - | - | - | - | - | - | - | - | - | - | - |
| 2 | Kaida(2011)^11^ | 15 | - | - | - | - | - | - | - | - | - | - | - | - |
| 3 | Ikeda(2012)^12^ | 55 | - | - | - | - | - | - | - | - | - | - | - | - |
| 4 | Linsuwanon(2012)^13^ | 25 | 9(36) | 1(4) | - | 5(20) | 3(12) | - | - | - | - | - | - |  |
| 5 | Imamura(2013)^14^ | 9 | - | - | - | - | - | - | - | - | - | - | - | - |
| 6 | Lu(2014)^15^ | 7 | 5(71.43) | 4(57.14) | - | 1(14.29) | - | - | - | - | 1(14.29) | - | - | - |
| 7 | Furuse(2015)^16^ | 20 | 3(15) | 3(15) | - | - | - | - | - | - | - | - | - | - |
| 8 | Xiao(2015)^17^ | 19 | 11(57.89) | 5(26.32) | - | 2(10.53) | - | - | 2(10.53) | - | 1(5.26) | - | - | - |
| 9 | Kaida(2016)^18^ | 18 | 3(16.67) | - | 1(5.56) | - | - | 2(11.11) | - | - | - | - | - | - |
| 10 | Thongpan(2016)^20^ | 5 | 0(0) | - | - | - | - | - | - | - | - | - | - | - |
| 11 | Zhang(2016)^21^ | 11 | 0(0) | - | - | - | - | - | - | - | - | - | - | - |
| 12 | Lam(2016)^19^ | 24 | - | - | - | - | - | - | - | - | - | - | - | - |
| 13 | Itagaki(2017)^22^ | 79 | 10(12.66) | - | 2(2.53) | - | - | - | 1(1.27) | 1(1.27) | - | - | 1(1.27) | 1(1.27) |
| 14 | Shen(2019)^23^ | 20 | 1(5) | - | - | - | - | - | - | - | - | - | - | - |
| 15 | Hasuwa(2020)^24^ | 10 | 1(10) | - | 1(10) | - | - | - | - | - | - | - | - | - |
| 16 | Mozhgani(2020)^25^ | 34 | - | - | - | - | - | - | - | - | - | - | - | - |
| 17 | Tang(2021)^26^ | 17 | 3(17.65) | - | - | - | - | - | - | - | - | 1(5.88) | - | - |
| 18 | Li(2023)^27^ | 10 | 2(20) | 1(10) | - | 1(10) | - | - | - | - | - | - | - | - |
| 19 | Ikuse(2024)^28^ | 42 | 34(80.95) | 25(59.52) | 10(23.81) | 3(7.14) | 1(2.38) | 5(11.9) | - | - | 1(2.38) | - | - | - |
| 20 | Li(2024)^29^ | 9 | 7(77.78) | 1(11.11) | 2(22.22) | - | - | - | 1(11.11) | 1(11.11) | - | - | - | - |

**Supplementary Table 6** The coinfection of children with EV-D68 analyzed by meta-analysis.

| **ID** | **Study** | | **N** | **n (%)** | | | | | | | | | | | | | | | | | | | | | | | | | |  |
| --- | --- | --- | --- | --- | --- | --- | --- | --- | --- | --- | --- | --- | --- | --- | --- | --- | --- | --- | --- | --- | --- | --- | --- | --- | --- | --- | --- | --- | --- | --- |
|  |  |  |  | **CMV** | | **EBV** | | ***MP*** | | ***CP*** | | ***SP*** | | ***HI*** | | ***MC*** | | ***EC*** | | ***MTB*** | | ***Fungus*** | | ***BP*** | | ***SA*** | | ***PA*** | |  |
| 1 | Imamura(2011)^10^ | 21 | | | - | | - | | - | | - | | - | | - | | - | | - | | - | | - | | - | | - | | - | |
| 2 | Kaida(2011)^11^ | 15 | | | - | | - | | - | | - | | - | | - | | - | | - | | - | | - | | - | | - | | - | |
| 3 | Ikeda(2012)^12^ | 55 | | | - | | - | | - | | - | | - | | - | | - | | - | | - | | - | | - | | - | | - | |
| 4 | Linsuwanon(2012)^13^ | 25 | | | - | | - | | - | | - | | - | | - | | - | | - | | - | | - | | - | | - | | - | |
| 5 | Imamura(2013)^14^ | 9 | | | - | | - | | - | | - | | - | | - | | - | | - | | - | | - | | - | | - | | - | |
| 6 | Lu(2014)^15^ | 7 | | | 1(14.29) | | 1(14.29) | | 1(14.29) | | 1(14.29) | | 1(14.29) | | - | | - | | - | | - | | - | | - | | - | | - | |
| 7 | Furuse(2015)^16^ | 20 | | | - | | - | | - | | - | | - | | - | | - | | - | | - | | - | | - | | - | | - | |
| 8 | Xiao(2015)^17^ | 19 | | | - | | - | | 1(5.26) | | - | | 2(10.53) | | 1(5.26) | | 3(15.79) | | 1(5.26) | |  | |  | |  | |  | |  | |
| 9 | Kaida(2016)^18^ | 18 | | | - | | - | | - | | - | | - | | - | | - | | - | | - | | - | | - | | - | | - | |
| 10 | Thongpan(2016)^20^ | 5 | | | - | | - | | - | | - | | - | | - | | - | | - | | - | | - | | - | | - | | - | |
| 11 | Zhang(2016)^21^ | 11 | | | - | | - | | - | | - | | - | | - | | - | | - | | - | | - | | - | | - | | - | |
| 12 | Lam(2016)^19^ | 24 | | | - | | - | | - | | - | | - | | - | | - | | - | | - | | - | | - | | - | | - | |
| 13 | Itagaki(2017)^22^ | 79 | | | 4(5.06) | | - | | - | | - | | - | | - | | - | | - | | - | | - | | - | | - | | - | |
| 14 | Shen(2019)^23^ | 20 | | | - | | - | | 1(5) | | - | | - | | - | | - | | - | | - | | - | | - | | - | | - | |
| 15 | Hasuwa(2020)^24^ | 10 | | | - | | - | | - | | - | | - | | - | | - | | - | | - | | - | | - | | - | | - | |
| 16 | Mozhgani(2020)^25^ | 34 | | | - | | - | | - | | - | | - | | - | | - | | - | | - | | - | | - | | - | | - | |
| 17 | Tang(2021)^26^ | 17 | | | - | | - | | 2(11.76) | | - | | - | | - | | - | | - | | - | | 1(5.88) | | - | | - | | - | |
| 18 | Li(2023)^27^ | 10 | | | - | | - | | - | | - | | - | | - | | - | | - | | - | | - | | - | | - | | - | |
| 19 | Ikuse(2024)^28^ | 42 | | | - | | - | | - | | - | | - | | - | | - | | - | | - | | - | | - | | - | | - | |
| 20 | Li(2024)^29^ | 9 | | | - | | - | | 2(22.22) | | - | | - | | - | | - | | - | | 1（11.11） | | - | | 1（11.11） | | 1（11.11） | | 1（11.11） | |

**Supplementary Table** 6 The coinfection of children with EV-D68 analyzed by meta-analysis(continued).

AdV, adenovirus; HCoV, human coronavirus; CMV, cytomegalovirus; HMPV, human metapneumovirus; HPIV, human parainfluenza virus type; BoV, Bocavirus; Flu, influenza virus; RV, rhinovirus; RSV, respiratory syncytial virus; EBV, Epstein - Barr virus; Cox B4, coxsackievirus B4; CP, *Chlamydia pneumoniae*; MP, *Mycoplasma pneumoniae*; SP, *Streptococcus pneumoniae*; BP, *Bordetella pertussis*; SA, *Staphylococcus aureus*; PA, *Pseudomonas aeruginosa*; MTB, *Mycobacterium tuberculosis*; HI, *Haemophilus influenzae*; MC, *Moraxella catarrhalis*; EC, *Enterobacter cloacae*.

**Supplementary Figure 1**Yearly detection rate between 2007-2019.

**
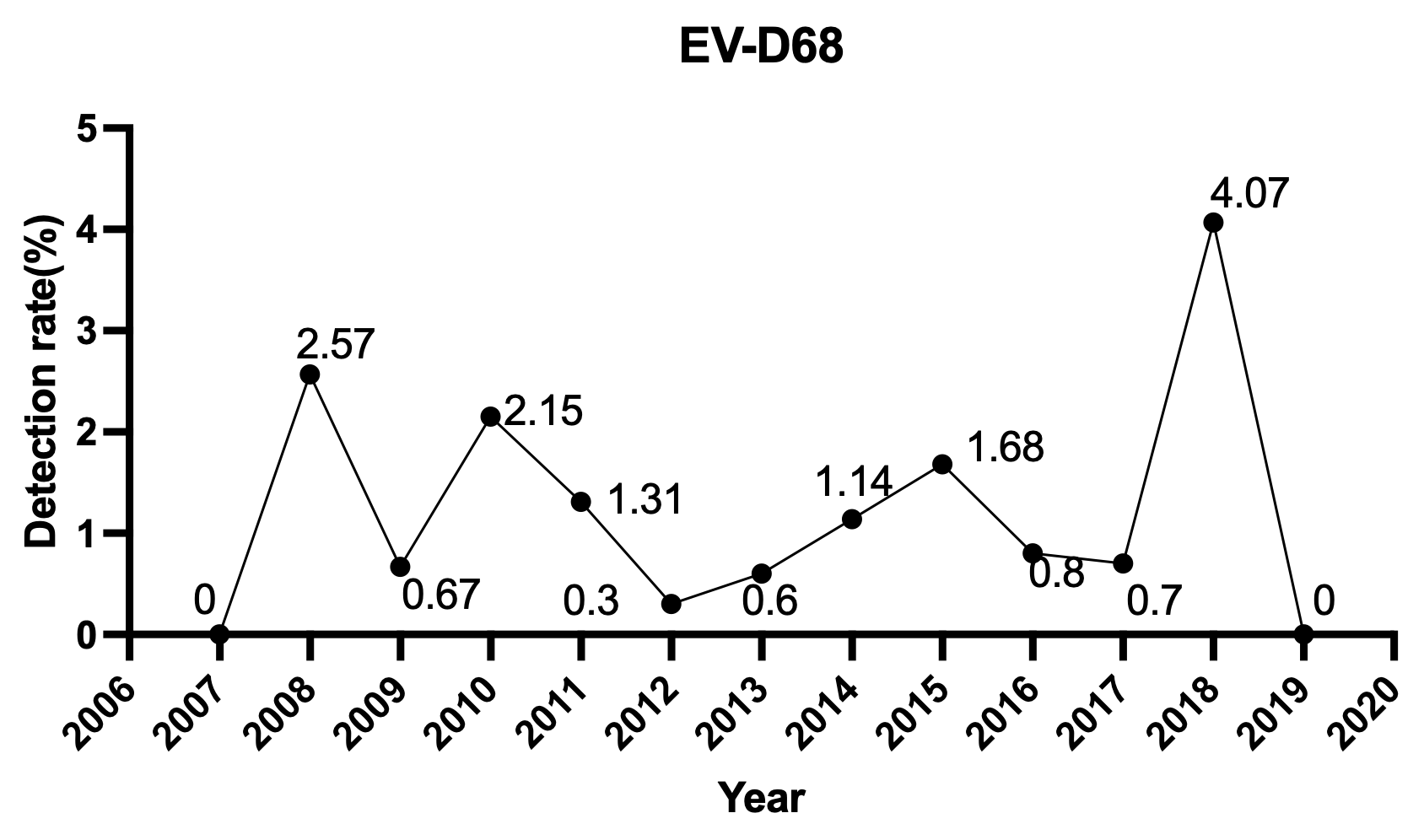
**

**Supplementary Figure 2** Begg’s test for the incidence of EV-D68-associated respiratory symptoms in Asian children.


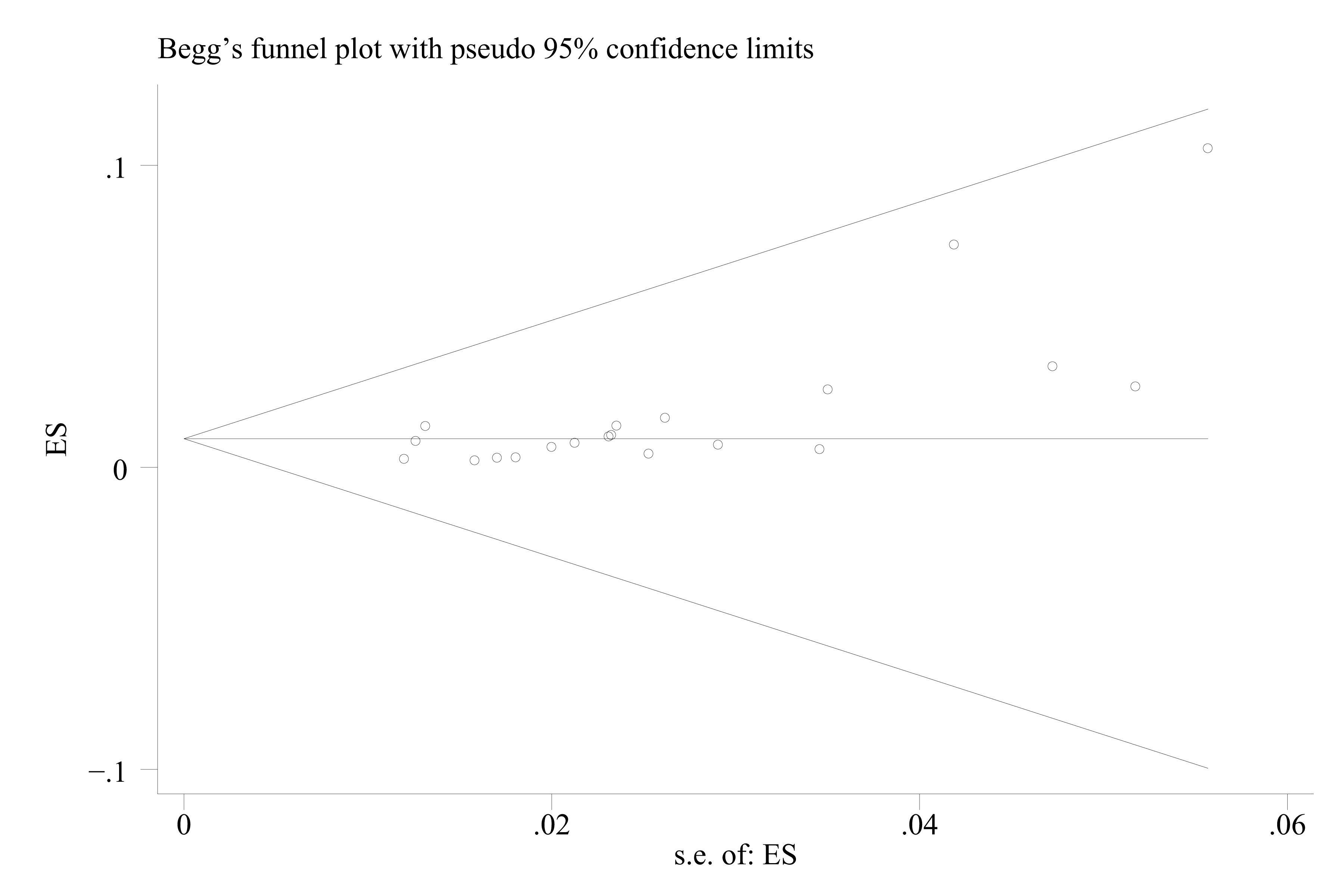


**Supplementary Figure 3** Funnel plot for publications about enterovirus D68 infection.


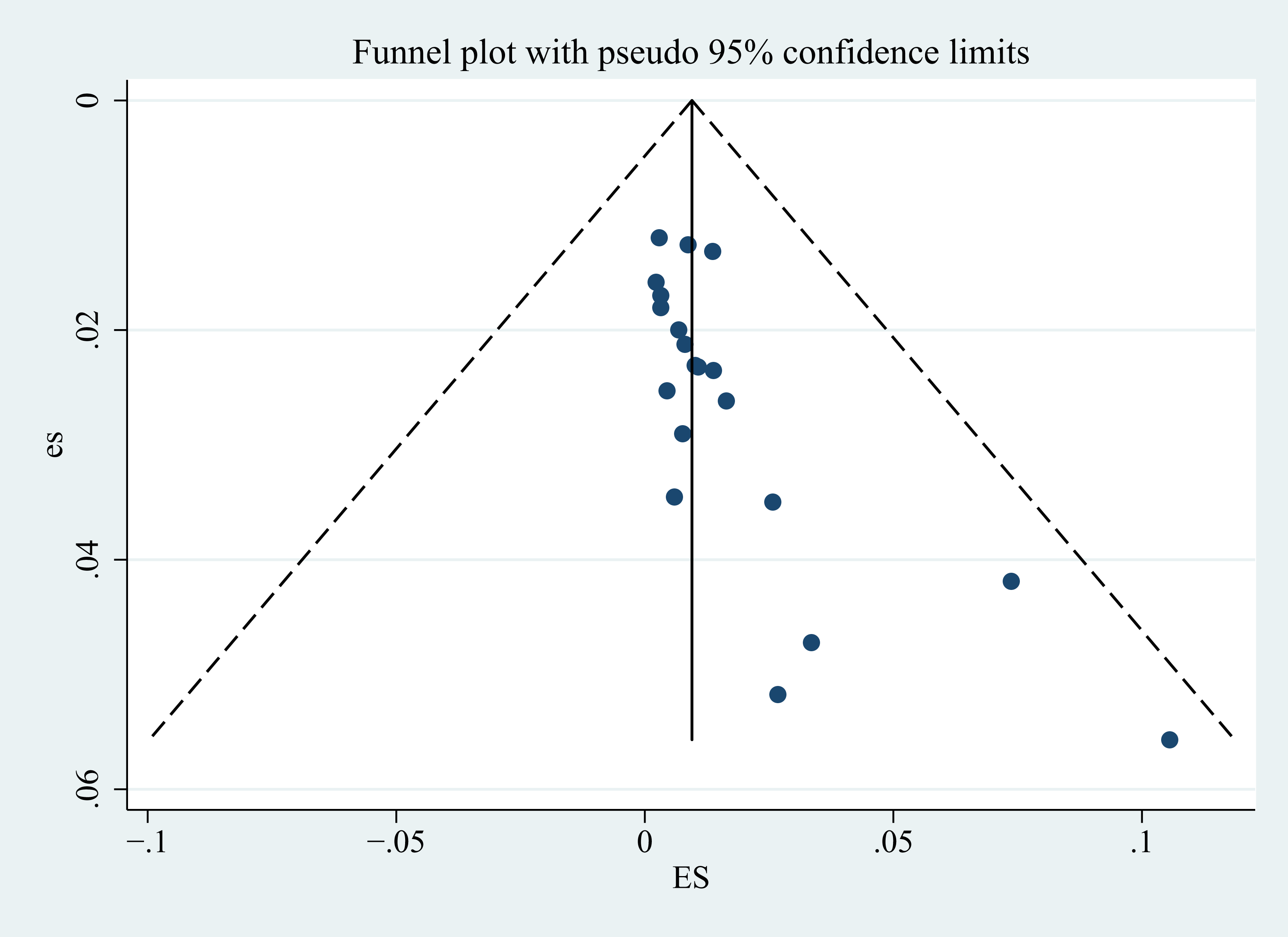

Supplement: Supplementary file 1 [file mmc1.docx]
